# Supplementary material for: A TrkB agonist prodrug prevents bone loss via inhibiting asparagine endopeptidase and increasing osteoprotegerin
Source: Nat Commun. 2022 Aug 16;13:4820. doi: 10.1038/s41467-022-32435-5 (PMC9381595; doi:10.1038/s41467-022-32435-5)
Supplement: Supplementary file 1 — Supplementary Information [file 41467_2022_32435_MOESM1_ESM.pdf]

Supplementary Figure 1

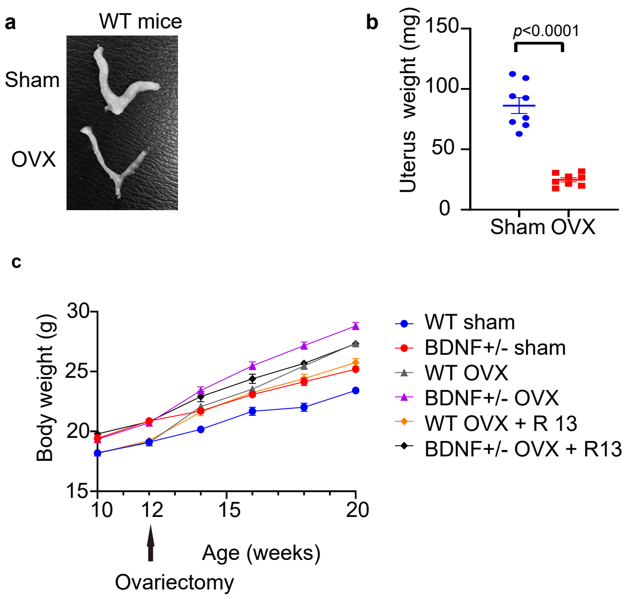

Supplementary Figure 1. Ovariectomy induced uterus atrophy in wild type mice.
(a) The uterus morphology of the wild-type mice (WT) with (OVX) or without (Sham) ovariectomy. (b) Uterus weight of the wild-type mice with (OVX) or without (Sham) ovariectomy. Data are shown as mean ± SEM, n = 8 mice per group, Two-tailed unpaired t-test. (c) Body weight of WT and BDNF +/- mice after sham or ovariectomy surgery followed by treatment with R13 or vehicle. Data are shown as mean ± SEM, n = 8 mice per group.

Supplementary Figure 2

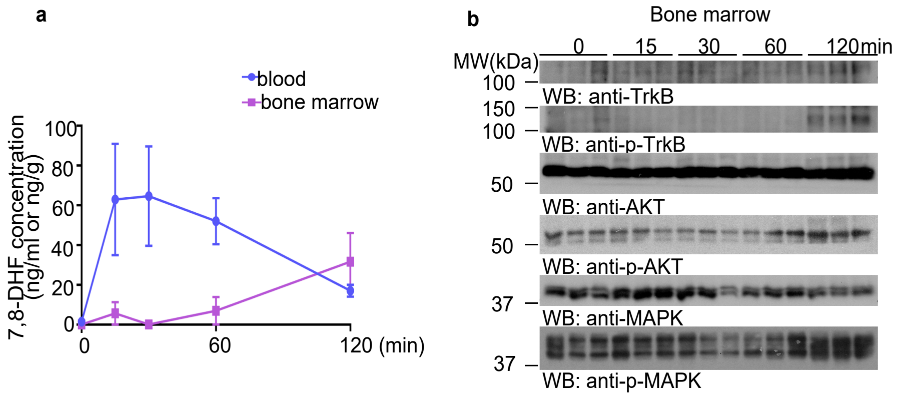

Supplementary Figure 2. In vivo PK study of R13 and PD/PK relationship.
(a) In vivo PK study. 2 months old female mice were treated with R13 (21.8mg/kg) by oral gavage, and the blood and bone marrow samples were collected at different time points (0, 15, 30, 60, 120 min) after R13 treatment (3 mice each group). 7,8-DHF concentration in the plasma (ng/ml) and bone marrow (ng/g) samples were quantitatively analyzed by LC-MS/MS. Data are shown as mean ± SEM, n = 3 mice per group. (b) Western blot showing the phosphorylation of TrkB, AKT and MAPK in bone marrow of the mice.

Supplementary Figure 3

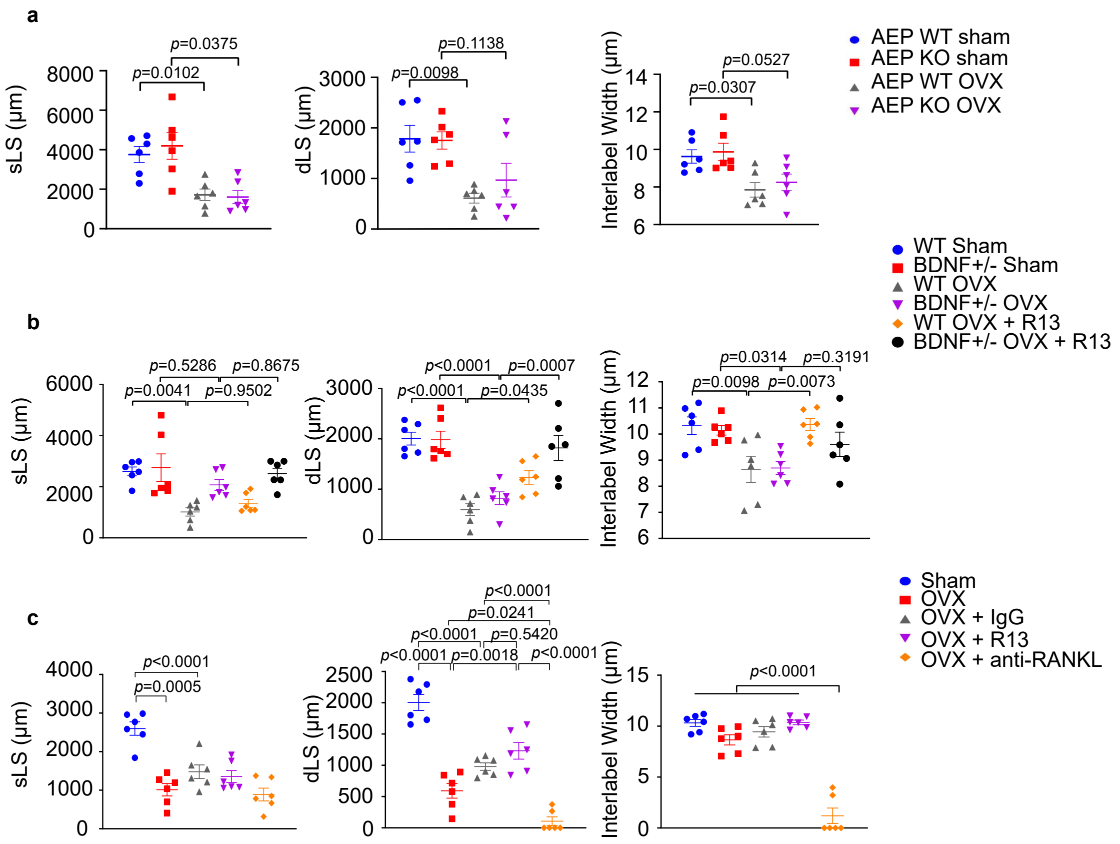

Supplementary Figure 3. Single- and double labelled surface and inter-label thickness data of the mice.
(a) sLS (single labelled surface), dLS (double labelled surface) and inter-label width of the femur bone in AEP WT or KO mice with sham or ovariectomy. (b) sLS, dLS and inter-label width of the femur bone in WT or BDNF+/- mice with sham or ovariectomy following R13 treatment or not. (c) sLS, dLS and inter-label width of the femur bone in WT with or without OVX followed by R13 or anti-Rankl antibody treatment. Data are shown as mean ± SEM, n = 6 mice per group, one-way ANOVA.

Supplementary Figure 4

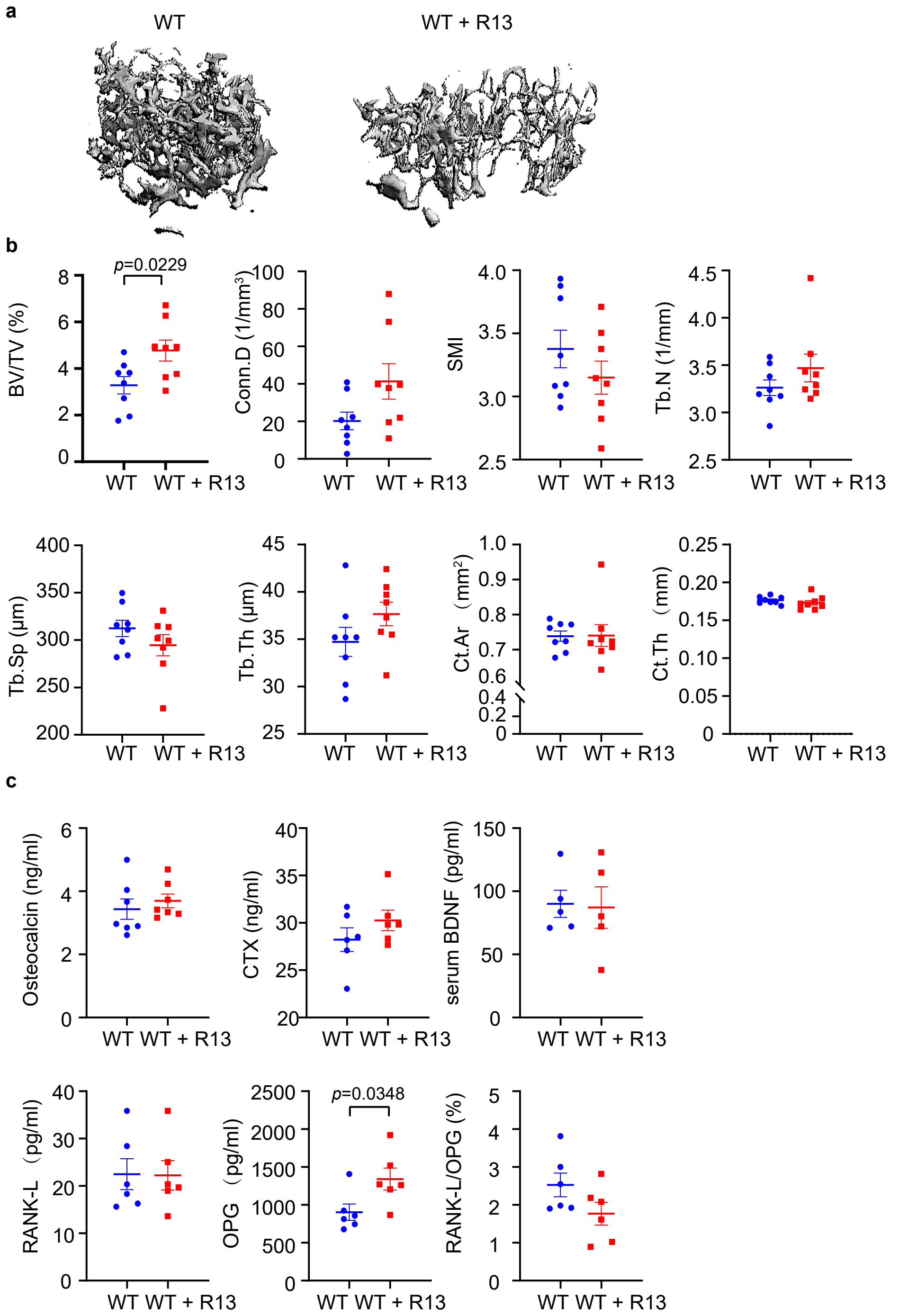

Supplementary Figure 4. R13 increases trabecular bone density in wild-type mice.
WT mice were treated with or without R13 (21.8 mg/kg) for 8 weeks (6 days per week) by oral gavage at 12 weeks old. Femoral bone structures were assessed by in vitro μCT. (a) Images of the femoral indices of trabecular bone structure measured by in vitro μCT scan. (b) R13 increases bone volume fraction in WT mice. μCT scanning measurements of trabecular bone volume fraction (BV/TV), Conn.D., Structure model index (SMI), Trabecular number (Tb.N), Trabecular spacing (Tb.Sp), trabecular thickness (Tb.Th), Cortical area (Ct.Ar) and average cortical thickness (Ct.Th). There's significant difference between WT and WT+ R13 mice in BV/TV (p = 0.0229), but there's no significant difference between WT and WT+ R13 mice in Conn.D. (p = 0.0649), SMI (p = 0.2728), Tb.N (p = 0.2336), Tb.Sp (p = 0.2336), Tb.Th (p = 0.1593), Ct.Ar (p = 0.9636) and Ct.Th (p = 0.3615). Data are shown as mean ± SEM, n = 8 mice per group, Two-tailed unpaired t-test. (c) Serum levels of Osteocalcin, CTX, RANK-L, OPG, RANK-L/OPG ratio and serum BDNF levels. There's significant difference between WT and WT+ R13 mice in OPG level (p = 0.0348), but there's no significant difference between WT and WT+ R13 mice in osteocalcin (p = 0.5139), CTX (p = 0.2449), RANK-L (p = 0.9612), RANK-L/OPG ratio (p = 0.111) and BNDF (p = 0.8826). Data are shown as mean ± SEM, n = 5 mice per group for BDNF measurement, n=6 mice per group for CTX measurement or n=7 mice per group for osteocalcin, RANKL and OPG measurement, Two-tailed unpaired t-test.

Supplementary figure 5

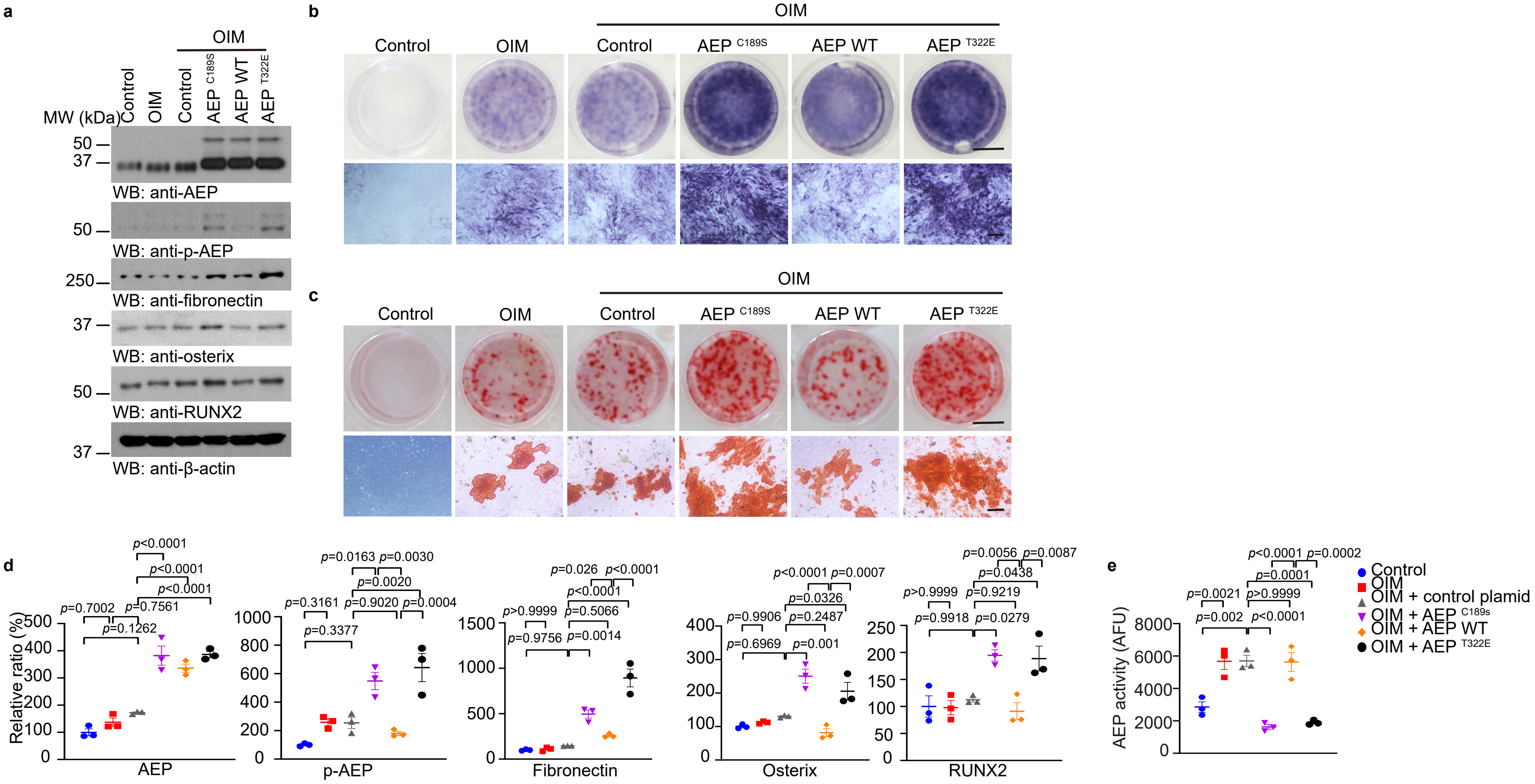

Supplementary Figure 5. Blocking AEP activity promotes MC3T3-E4 cell differentiation and mineralization.
MC3T3 cells were cultured in OIM and transfected with or without AEP C189S plasmid, AEP WT plasmid and AEP T322E plasmid. (a) Western blot showed the expression of AEP, p-AEP, fibronectin, osterix and RUNX2. (b) Representative images of ALP staining positive cells in MC3T3 cells cultured in OIM with or without AEP C189S plasmid, AEP WT plasmid and AEP T322E plasmid transfection for 14 days (n=3 independent experiments) (Scale bar, 5mm (upper panel), 200 µm (lower panel)). (c) Representative images of Alizarin Red S mediated calcium staining in MC3T3-E4 cells treated with or without AEP C189S plasmid, AEP WT plasmid and AEP T322E plasmid transfection for 21 days (n=3 independent experiments) (Scale bar, 5mm (upper panel), 200 µm (lower panel)). (d) Quantification of the relative protein levels of AEP, p-AEP, fibronectin, osterix and runx2. Data are shown as mean ± SEM of 3 biologically independent experiments, one-way ANOVA. (e) AEP enzymatic activity assay. Data are shown as mean ± SEM of 3 biologically independent experiments, one-way ANOVA.

Supplementary Figure 6

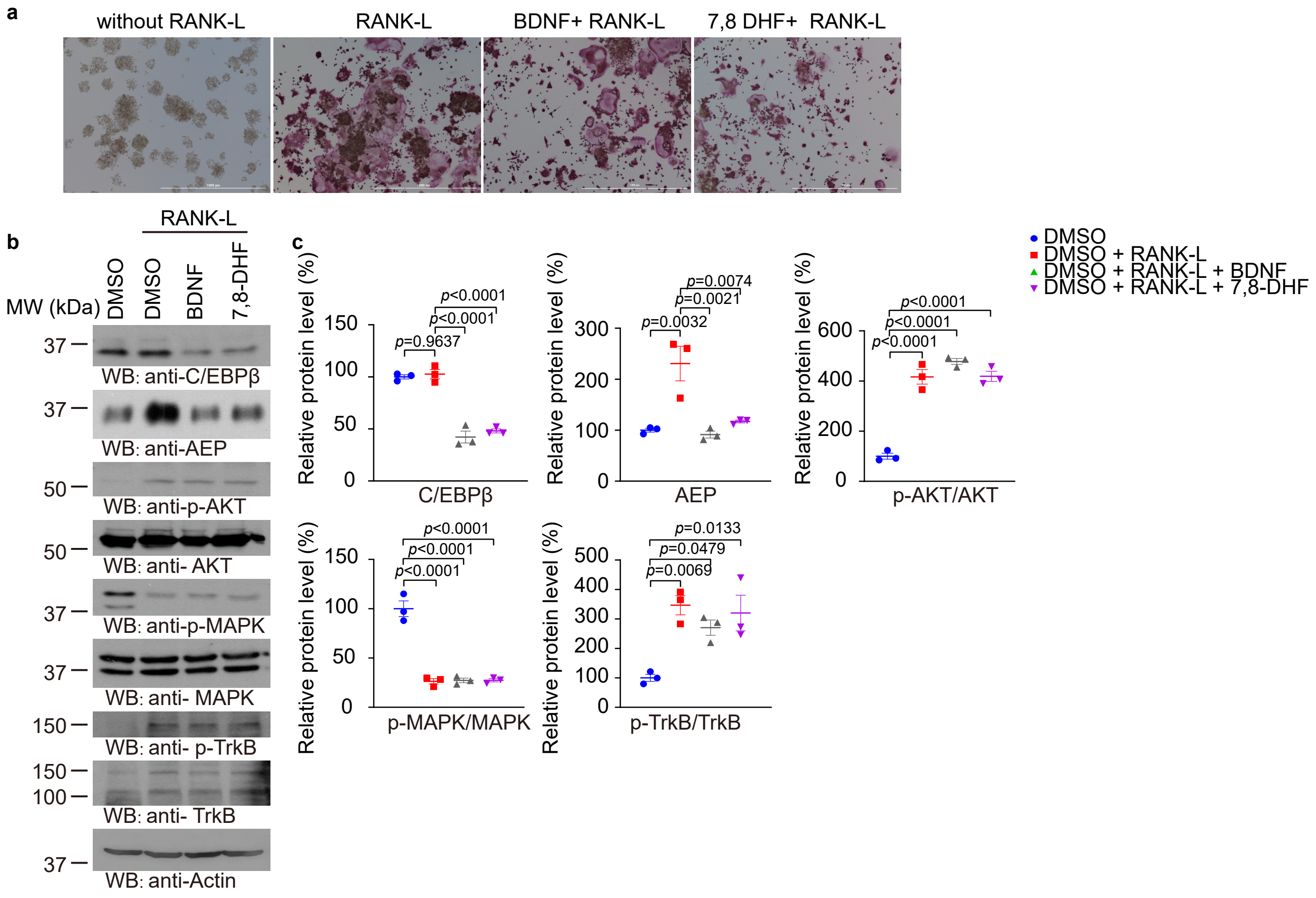

Supplementary Figure 6. 7,8-DHF inhibits RANK-L-induced RAW264.7 osteoclastogenesis.
(a) Representative images of TRAP staining of RAW 264.7 cells induced by RANKL with or without BDNF or 7,8-DHF for 4 days (n=3 independent experiments) (Scale bar, 1mm). (b& c) Western blotting showed that BDNF and 7,8-DHF inhibited C/EBPβ/AEP pathway, and activated p-TrkB and p-MAPK and p-AKT signaling. Data are shown as mean ± SEM of 3 biologically independent experiments, one-way ANOVA.
