# Supplementary material for: A TrkB agonist prodrug prevents bone loss via inhibiting asparagine endopeptidase and increasing osteoprotegerin
Source: Nat Commun. 2022 Aug 16;13:4820. doi: 10.1038/s41467-022-32435-5 (PMC9381595; doi:10.1038/s41467-022-32435-5)
Supplement: Supplementary file 2 — Reporting Summary [file 41467_2022_32435_MOESM2_ESM.pdf]

nature portfolio

Corresponding author(s): Keqiang Ye

Last updated by author(s): Jun 3, 2022

Reporting Summary

Nature Portfolio wishes to improve the reproducibility of the work that we publish. This form provides structure for consistency and transparency in reporting. For further information on Nature Portfolio policies, see our Editorial Policies and the Editorial Policy Checklist.

Statistics

For all statistical analyses, confirm that the following items are present in the figure legend, table legend, main text, or Methods section.

n/a	Confirmed	
☐	☒	The exact sample size (n) for each experimental group/condition, given as a discrete number and unit of measurement
☐	☒	A statement on whether measurements were taken from distinct samples or whether the same sample was measured repeatedly
☐	☒	The statistical test(s) used AND whether they are one- or two-sided<br>*Only common tests should be described solely by name; describe more complex techniques in the Methods section.*
☒	☐	A description of all covariates tested
☐	☒	A description of any assumptions or corrections, such as tests of normality and adjustment for multiple comparisons
☐	☒	A full description of the statistical parameters including central tendency (e.g. means) or other basic estimates (e.g. regression coefficient) AND variation (e.g. standard deviation) or associated estimates of uncertainty (e.g. confidence intervals)
☐	☒	For null hypothesis testing, the test statistic (e.g. F, t, r) with confidence intervals, effect sizes, degrees of freedom and P value noted<br>*Give P values as exact values whenever suitable.*
☒	☐	For Bayesian analysis, information on the choice of priors and Markov chain Monte Carlo settings
☒	☐	For hierarchical and complex designs, identification of the appropriate level for tests and full reporting of outcomes
☒	☐	Estimates of effect sizes (e.g. Cohen's d, Pearson's r), indicating how they were calculated

Our web collection on statistics for biologists contains articles on many of the points above.

Software and code

Policy information about availability of computer code

Data collection	For bone µCT measurements, the data was captured and analyzed by a Scanco µCT-40 scanner. Histomorphometry was done using the Bioquant Image Analysis System. All real-time PCR reactions were performed using the ABI 7500-Fast Real-Time PCR System, and the data were collected from the ABI 7500 Software Version v2.3.
Data analysis	Graphpad Prism v.9 was used to analyze the data. This is stated under "Statistics and reproducibility", in the Methods section of the manuscript.

For manuscripts utilizing custom algorithms or software that are central to the research but not yet described in published literature, software must be made available to editors and reviewers. We strongly encourage code deposition in a community repository (e.g. GitHub). See the Nature Portfolio guidelines for submitting code & software for further information.

Data

Policy information about availability of data

All manuscripts must include a data availability statement. This statement should provide the following information, where applicable:

- Accession codes, unique identifiers, or web links for publicly available datasets
- A description of any restrictions on data availability
- For clinical datasets or third party data, please ensure that the statement adheres to our policy

All raw data of Western blot image in this study are included in the study. The original source data used to generate graphs in each of the figures and supplementary figures are provided as Microsoft Excel data sheet files. There're no restrictions on data availability. Source data are provided with this paper.

Field-specific reporting

Please select the one below that is the best fit for your research. If you are not sure, read the appropriate sections before making your selection.

[x] Life sciences   [ ] Behavioural & social sciences   [ ] Ecological, evolutionary & environmental sciences

For a reference copy of the document with all sections, see nature.com/documents/nr-reporting-summary-flat.pdf

Life sciences study design

All studies must disclose on these points even when the disclosure is negative.

Sample size	No statistical methods were used to calculate sample size. Instead, sample sizes were determined on the basis of previous studies by Dr. Ye's and other groups [PMID: 31980603]. For western blotting, PCR, ELISA and immunohistochemistry, samples were derived from at least three animals of 3 independent experiments.
Data exclusions	No data were excluded.
Replication	For each experiment, the number of biological replicates is reported in the figure legend.
Randomization	Mice were randomly selected to be assigned into separate group. All mice were of the same species, sex and age. For analysis of the H&E, TRAP staining and quantitative bone histomorphometry were taken from randomly mice.
Blinding	For bone µCT measurements, the data was captured by the technicians in Dr. Pacific's Lab who were blind to the sample information. Histomorphometry were conducted in the University of Alabama at Birmingham Center for Metabolic Bone Disease-Histomorphometry and Molecular Analysis Core Laboratory by technicians who were unaware of the mouse groups. Investigators were blinded to the group allocation during the data collection and blinded to sample identity for the analysis of immunohistochemistry and western blotting.

Reporting for specific materials, systems and methods

We require information from authors about some types of materials, experimental systems and methods used in many studies. Here, indicate whether each material, system or method listed is relevant to your study. If you are not sure if a list item applies to your research, read the appropriate section before selecting a response.

Materials & experimental systems

n/a	Involved in the study
[ ]	[x] Antibodies
[ ]	[x] Eukaryotic cell lines
[x]	[ ] Palaeontology and archaeology
[ ]	[x] Animals and other organisms
[x]	[ ] Human research participants
[x]	[ ] Clinical data
[x]	[ ] Dual use research of concern

Methods

n/a	Involved in the study
[x]	[ ] ChIP-seq
[x]	[ ] Flow cytometry
[x]	[ ] MRI-based neuroimaging

Antibodies

Antibodies used	Antibodies to C/EBPβ (mouse monoclonal, catalog#: sc-7962, clone HT-7), RANKL (mouse monoclonal, catalog#: sc-377079, clone G-1), OPG (mouse monoclonal, catalog#: sc-390518, E-10), osterix (mouse monoclonal, catalog#: sc-393325, F-3) and RUNX2 (mouse monoclonal, catalog#: sc-101145, 27-K) was from Santa Cruz; Antibodies to Legumain (rabbit monoclonal, catalog#: 93627, clone D6S4H), p-C/EBPβ (rabbit polyclonal, catalog#: 3084s), AKT (rabbit monoclonal, catalog#: 4691s, clone C67E7), p-AKTS473 (rabbit polyclonal, catalog#: 9271s), MAPK (rabbit polyclonal, catalog#: 9102s), p-MAPK (Thr202/Tyr204, mouse monoclonal, catalog#: 9106s, clone E10), p-c-Jun (Ser73) (rabbit monoclonal, catalog#: 3270T), c-Jun (rabbit monoclonal, catalog#: 9165T, clone 60A8), CREB (rabbit monoclonal, catalog#: 9197T, clone 48H2) and p-CREB (rabbit monoclonal, catalog#: 9198T, clone 87G3) were purchased from Cell Signaling Technology; Antibody to TrkB (mouse monoclonal, catalog#: MAB397-500, clone#75133) was from R&D; Antibody to β-actin (mouse monoclonal, catalog#: A5316, clone AC-74) and Fibronectin (rabbit polyclonal, catalog#: F3648) were from Sigma-Aldrich; Rabbit polyclonal antibodies to p-AEP (T322) and p-TrkB (Tyr816) were developed in the Ye lab; anti-RANKL monoclonal antibody (rat monoclonal, catalog#: 510012, clone IK22/5) and anti-IgG antibody (catalog#: 401412) for mice treatment is obtained from Ichorbio.
Validation	(1) Antibody to C/EBPβ (H-7) detects C/EBPβ of mouse, rat and human origin by Western blotting, immunoprecipitation, immunofluorescence and immunohistochemistry (PMID: 29725016).<br>(2) Antibody to RANKL (G-1) detects RANKL of mouse, rat and human origin by Western blotting, immunoprecipitation, immunofluorescence, immunohistochemistry and elisa (PMID: # 32342860).<br>(3) Antibody to OPG (E-10) detects OPG of mouse, rat and human origin by Western blotting, immunoprecipitation, immunofluorescence and elisa (PMID: 29486220).

(4) Antibody to osterix (F-3) detects OSX of mouse, rat and human origin by Western blotting, immunoprecipitation, immunofluorescence and elisa (PMID: # 29414599).
(5) Antibody to RUNX2 (27-K) detects RUNX2 of mouse, rat and human origin by Western blotting, immunoprecipitation, immunofluorescence, immunohistochemistry and elisa.
(6) Antibody to Legumain (catalog#: 93627, clone D6S4H) interacts with human, mouse and rat proteins on immunofluorescence and Western blotting (PMID: 31793911).
(7) Antibody to Phospho-C/EBPβ (Thr235) detects endogenous levels of human LAP only when phosphorylated at Thr235, mouse and rat LAP only when phosphorylated at Thr188, and LIP only when phosphorylated at Thr37. This antibody does not cross-react with other phosphorylated C/EBPs.
(8) Antibody to AKT (catalog#: 4691s, clone C67E7) detects endogenous levels of human, mouse, rat and monkey total Akt protein, and does not cross react with other related proteins by Western blotting, immunofluorescence and other immune detection methods.
(9) The pAKTS473 (catalog#: 9271s) antibody detects endogenous levels of human, mouse, rat, hamster, monkey, cow and dog Akt1 only when phosphorylated at Ser473 (Western blotting and immunofluorescence). This antibody also recognizes Akt2 and Akt3 when phosphorylated at the corresponding residues, and does not recognize Akt phosphorylated at other sites, nor does it recognize phosphorylated forms of related kinases such as PKC or p70 S6 kinase.
(10) The MAPK (catalog#: 9102s) antibody has been validated for Western blotting and immunohistochemistry of human, mouse, rat, hamster, monkey, mink, cow and pig total p44/42 MAP kinase (Erk1/Erk2) protein. In some cell types, this antibody recognizes p44 MAPK more readily than p42 MAPK. The antibody does not recognize either JNK/SAPK or p38 MAP kinase.
(11) Antibody to p-44/42MAPK (catalog#: 9106s, clone E10) detects human, mouse, rat, hamster, monkey, mink, cow and pig p44 and p42 MAP kinases (Erkl and Erk2) by Western blotting, when dually phosphorylated at Thr202 and Tyr204 of Erk1 (Thr185 and Tyr187 of Erk2), but not singly phosphorylated at Thr202 or Tyr204. The antibody does not cross-react with the corresponding phosphorylated residues of either SAPK/JNK or p38 MAP kinase. This antibody may cross-react with an unknown cytoskeletal protein in some cell lines as visualized by immunofluorescence.
(12) Antibody to Phospho-c-Jun (Ser73) (catalog#: 3270T) detects human, mouse, rat, monkey and pig c-Jun only when phosphorylated at Ser73. This antibody may also recognize JunD phosphorylated at Ser100.
(13) Antibody to c-Jun (catalog#: 9165T, clone 60A8) detects human, mouse, rat, monkey endogenous levels of total c-Jun protein, regardless of phosphorylation state.
(14) Antibody to CREB (catalog#: 9197T, clone 48H2) detects Human, Mouse, Rat, Monkey, D. melanogaster endogenous levels of total CREB-1 protein. The antibody does not cross-react with other ATF/CREB family members. Non-specific staining of components along the retinotectal pathway was observed by immunofluorescence in fixed frozen mouse tissue.
(15) Antibody to Phospho-CREB (Ser133) (catalog#: 9198T, clone 87G3) detects human, mouse, rat endogenous levels of CREB only when phosphorylated at serine 133. The antibody also detects the phosphorylated form of the CREB-related protein, ATF-1.
(16) Antibody to TrkB (catalog#: MAB397-500, clone#75133) Detects human TrkB in direct ELISAs. Detects human, mouse, and rat TrkB in Western blots. In ELISAs and Western blots, no cross-reactivity with recombinant human (rh) TrkA, rhTrkC, or recombinant rat TrkA is observed.
(17) Antibody to β -actin (catalog#: A5316, clone AC-74) detects protein on Western blotting, immunofluorescence and immunohistochemistry in tissues from humans, rat, mouse, pig, dog, sheep and guinea pig.
(18) Antibody to Fibronectin (catalog#: F3648) shows no cross-reaction with laminin, vitronectin, collagen type IV, or chondroitin sulfate types A, B and C using a dot blot immunoassay.
(19) Antibodies to p-AEP (T322) and p-TrkB (Tyr816) were developed in the Ye lab and have been validated in multiple publications for Western blotting, immunohistochemistry and immunofluorescence of human, mouse and rat tissues [PMID: 28826672; PMID 25326800; PMID: 26549211; and PMID: 19592491].
(20) Anti-RANKL monoclonal antibody (catalog#: 510012, clone IK22/5) and anti-IgG antibody (catalog#: 401412) was used in multiple publications for block reaction in mice. [PMID: 18218689; PMID: 17371958]

Eukaryotic cell lines

Policy information about cell lines

Cell line source(s)	Murine MC3T3-E1 (subclone 4) cells and RAW 264.7 cells were obtained from American Type Culture Collection (ATCC, Manassas, VA, USA)
Authentication	Not authenticated.
Mycoplasma contamination	Not tested for Mycoplasma.
Commonly misidentified lines (See ICLAC register)	None.

Animals and other organisms

Policy information about studies involving animals; ARRIVE guidelines recommended for reporting animal research

Laboratory animals	BDNF+/- mice were obtained from Jackson Laboratory (MMRRC stock#002267). The AEP knockout mice on a mixed C57BL/6 and 129/Ola background were generated as reported (J Biol Chem. 2003 Aug 29) . All in vivo experiments were carried out in female. BDNF+/- mice, AEP WT and AEP knockout mice were bilaterally ovariectomized or sham operated at 12 weeks of age.
Wild animals	Female C57BL6/J wild-type mice obtained from Jackson Laboratory (MMRRC stock#000664), WT mice were bilaterally ovariectomized or sham operated at 12 weeks of age.

Field-collected samples	The study did not involve samples to be collected from the field.
Ethics oversight	The experiments were conducted according to the NIH animal care guidelines and Emory School of Medicine guidelines. The protocol was reviewed and approved by the Institutional Animal Care and Use Committee (IACUC) at Emory University.

Note that full information on the approval of the study protocol must also be provided in the manuscript.
